# Invisibility in global health: A case for disturbing bioethical frameworks

**DOI:** 10.12688/wellcomeopenres.19346.2

**Published:** 2023-12-14

**Authors:** Arsenii Alenichev, Halina Suwalowska, Marlyn C. Faure, Shu Hui Ng, Chelsea Modlin, Ilana Ambrogi, Jonathan D. Shaffer, Michael Parker, Patricia Kingori

**Affiliations:** 1 Ethox Centre, Wellcome Centre for Ethics and Humanities, Nuffield Department of Population Health, University of Oxford, Oxford, England, OX3 7LF, UK; 2 The Ethics Lab, Department of Medicine and Neuroscience Institute, University of Cape Town, Rondebosch, Cape Town, 7935, South Africa; 3 Monash University, Subang Jaya, Selangor, 45700, Malaysia; 4 Berman Institute of Bioethics, Johns Hopkins University, Baltimore, Maryland, 21205, USA; 5 Institute of Bioethics, Human Rights and Gender (ANIS), Brasilia, 70.094-971, Brazil; 6 PPGBIOS/ENSP, Fiocruz, Rio de Janeiro, RJ, 21040-900, Brazil; 7 Department of Sociology, The University of Vermont, Burlington, Vermont, 05405, USA

**Keywords:** global health, bioethics, invisibility, inequality, decolonization

## Abstract

In recent years, the global health community has increasingly reported the problem of ‘invisibility’: aspects of health and wellbeing, particularly amongst the world’s most marginalized and impoverished people, that are systematically overlooked and ignored by people and institutions in relative positions of power.

It is unclear how to realistically manage global health invisibility within bioethics and other social science disciplines and move forward. In this letter, we reflect on several case studies of invisibility experienced by people in Brazil, Malaysia, West Africa and other transnational contexts. Highlighting the complex nature of invisibility and its interconnectedness with social, political and economic issues and trends, we argue that while local and targeted interventions might provide relief and comfort locally, they will not be able to solve the underlying causes of invisibility.

Building from the shared lessons of case study presentations at an Oxford-Johns Hopkins Global Infectious Disease Ethics Collaborative (GLIDE), we argue that in dealing with an intersectional issue such as invisibility, twenty-first century global health bioethics could pursue a more ‘disturbing’ framework, challenging the narrow comforting solutions which take as a given the sociomaterial inequalities of the status quo. We highlight that comforting and disturbing bioethical frameworks should not be considered as opposing sides, but as two approaches working in tandem in order to achieve the internationally set global health milestones of providing better health and wellbeing for everyone. Insights from sociology, anthropology, postcolonial studies, history, feminist studies and other styles of critical reasoning have long been disturbing to grand narratives of people and their conditions. To rediscover the ethos of the WHO Alma Ata Declaration—a vision of “health for all by the year 2000”—these thinking tools will be necessary aids in developing cooperation and support beyond the narrow market logic that dominates the landscape of contemporary global health.

## Disclaimer

This text represents the views of the authors and does not necessarily reflect the views of the agencies or institutions with which they are affiliated.

## Encountering global health invisibility: a few empirical snapshots

The academic discipline of global health ^1^ ( Farmer *et al.*, 2013) is increasingly acknowledging ^2^ that many social, political and economic aspects of health often remain structurally overlooked, underappreciated, or ignored ( Chambers, 2010; Parry *et al.*, 2019; Rahman-Shepherd *et al.*, 2021; The Lancet Global Health, 2019; WHO, 2022). These problems are made invisible by the accreted, institutionalized ways of knowing and intervening on them that have constituted the field. The literature reports this ‘invisibility’ unfolding in unequal or otherwise divided environments marked by asymmetrical power relations, which characterizes most—if not all—global health contexts ( Davis, 2017; Harman, 2016; Mac-Seing *et al.*, 2019; The Lancet Global Health, 2019). This means that global health invisibility offers a sharp challenge but also an opportunity to extend theory and practice.

In 2022, the Oxford-Johns Hopkins Global Infectious Disease Ethics Collaborative (GLIDE) organized a forum examining invisibility across a variety of global health contexts. The aim was to gain diverse perspectives to shift attention from the question of ‘ *What* is invisibility?’ to ‘ *How* is invisibility made and unmade in practice?’ and ‘How should ideas of invisibility be addressed in policy?’ In this letter, we do not provide record of that meeting, but rather an analysis of shared empirics and an argument presented based on those presentations. We reflect on the invisibility case studies and suggest a distinction between ‘disturbing’ and ‘comforting’ global bioethics as both an analytic lens and a way of doing bioethics that can productively work on the stubborn problem of invisibility, even the invisibility of power itself operating within bioethics ( Muntaner *et al.*, 2012). Moving forward and looking back, we argue that the ethos of the Alma Ata declaration ( WHO, 1978) should be rediscovered and reinvigorated for tackling global health invisibility and disturbing the comforting yet limited solutions to chronic intersectional issues.

In this section, we briefly introduce five examples of global health invisibility that reveal only the tip of the iceberg, with far more serious problems lying underneath. Firstly, in Malaysia, since the turn of the century, invisibility has unfolded as an institutionalized practice, wherein undocumented migrants, subjected to a spectrum of health problems and especially mental health issues, were not identifiable in any registries, and nor did their health issues in effect ‘exist’, effectively turning invisibility into a local determinant of health and wellbeing. Secondly, In Brazil, in the context of the response to Covid-19, layers of invisibility came to the fore with the lack of gender-specific policies and institutionalized processes that oversimplified continuums of inequalities and rendered vulnerable populations invisible.

Thirdly, we discussed how internationally praised collaborative partnerships for Ebola research in West Africa contained invisible forms of precarity stemming from a dependency on Global North-based institutional funding. As a result, the entire local research and health care systems relied on its presence and constant flow, creating cycles of unresolvable and unsustainable issues. This unseen phenomenon was in sharp contrast to the formal rhetoric of building local capacity and equal partnerships between the Global North and South. Moreover, we discussed how the US-led, North-South collaborative partnerships on global health and bioethics contained hidden layers of inequality that proliferated despite attempts to achieve benchmarks of fairness, empowerment and egalitarianism. Finally, zooming out at the international level, invisibility was traced with regard to the unseen politics of death and dying, spanning global health as a whole. There are millions of dead bodies across the globe that remain unidentified, and this number is steadily increasing due to humanitarian disasters, infectious disease outbreaks and mass migrations. In such contexts, people from poorer and marginalized backgrounds are likely to be invisible in their death and dying.

## Rethinking global bioethics: the case for disturbing and comforting methods

The collective discussion of such case studies brought up a key theme; namely, that topical and narrow solutions to invisibility will likely not be able to address the root causes of invisibility, however, they might be able to alleviate tensions locally. We further develop this idea and suggest that in order to address a complex issue such as global health invisibility it might be useful to maintain and utilize the analytic distinction between what we term ‘comforting’ and ‘disturbing’ global health bioethics. We argue that if it is true that invisibility is a function of coloniality within bioethics and global health more broadly, a two-pronged strategy of comforting and disturbing bioethics must be pursued, lest global health bioethics reproduce regimes of visibility and invisibility and the structural injustices they maintain.

The notion of visibility’ or ‘invisibility’ as a category of analysis within the social sciences ( Brighenti, 2007) and studies of global health is not new ( Brandt, 2013; Brown *et al.*, 2012). Visibility has been studied as a field of power, condensed and deployed by states and academic disciplines to manage and control populations, social problems, and expert knowledge respectively ( Foucault, 1972; Foucault, 1973; Scott, 1998). Within global health, the notion of visibility has been theorized as the political construction and mobilization of justificatory and explanatory metrics, quantitative and qualitative facts in global health governance ( Adams *et al.*, 2016; Benton, 2012). Subaltern and postcolonial studies have long emphasized the partial, positioned, and power-laden relationships that enable certain views, conceptions, and thus unequal knowledge and often reproduction of dominating transnational relationships and inequality ( Grosfoguel, 2002; Grosfoguel, 2007). Scholarly claims of “invisibility” in global health may also be related to the linked political and moral economies of ignorance ( Kourany & Carrier, 2020), secrecy, concealment, and hiding ( Jones, 2014; Scott, 2009: 179–219), as well as social hierarchies of recognition and their relationship to legibility and care ( Benton, 2015: 97–113). As this diverse social scientific literature demonstrates, the concepts of visibility and invisibility are inseparable from a normatively contested and highly unequal political economy that shapes the terrain of global health practice. A bioethics in and of global health must take note.

Reflecting on the snapshots of the case studies, we suggest that comforting bioethics builds upon the premise of creating new forms of normative engagement based on amendments to existing power structures, while effectively maintaining the status quo. In doing so, comforting bioethics effectively reproduces the idea of ethical commensurability, progress and Whig historiography ( Lerner & Caplan, 2016) in suggesting that ethicality is being gradually accumulated over time. Hence, the solutions are ‘comforting’ as they address issues locally and do not intend to fundamentally challenge the features of the entrenched power structures and socio-political determinants of health inequities.

While we are sympathetic for the need for psychic and moral comfort in a turbulent and dangerous world, we nevertheless cannot ignore the fact that all of the cases above, one way or another, are linked to global patterns of economies of material extraction, violent labor conditions, and socioeconomic distress and disparity that exists beyond the traditional remit of bioethics and global health analyses. Colonial history, the global acceleration of neoliberalism, and the neo-colonial integration of the Global South into global markets of unequal exchange, as well as patriarchal power dynamics, all play a role in creating and maintaining invisibility that we can no longer afford to overlook and oversimplify. Disturbingly, this could suggest that isolated attempts to address global health invisibility—without addressing or at least acknowledging the core issues and overarching patterns—risk misrepresenting the sheer magnitude of the problem. This, in turn, risks producing ‘cruel optimism’ ( Berlant, 2011): a social change that is simultaneously desired but is not attainable within a given socioeconomic system and the solutions it offers.

While comforting bioethics is well represented in applied bioethics and global health, we argue that the international community should practice more disturbing bioethics in order to achieve the internationally-postulated milestones for global health in the 21st century. Disturbing bioethics offers a different proposal: that ethicality can be achieved when power structures are challenged and reassembled in a structurally competent way. This simple yet crucial analytic point creates a justification for disturbing the comforting solutions and the sentimental morality they might entail. We suggest that disturbing bioethics could be enacted by drawing on insights from anthropology, sociology, studies of international development and globalization, postcolonial studies, community organizing and social movement studies and other styles of reasoning that have a record of disrupting power structures and challenging grand assumptions about people and their conditions. Disturbing bioethics, in essence, is an exercise in empirical reflexivity and a reminder that the contemporary world is marked by contingency, crisis and precarity. Such processes have been accelerating in recent years and now, without exaggeration, they pose an existential threat to humanity as a whole.

As a result, we encounter a timely and uneasy question for global health and bioethics: What are the ethical implications for offering reassuring and comforting interventions and frameworks to intersectional issues such as invisibility, knowing that it will, after being supposedly addressed in one given context, simply manifest somewhere else? 

## Disturbing guidance: The Alma Ata declaration revisited

Fortunately, we already have a milestone document that supports the claims of disturbing bioethics: WHO’s Alma Ata declaration (1978), which formulated health care as a fundamental human right that care should be available to all people regardless of their socio-economic status. The declaration called for a more horizontal approach to the structuring of infrastructures providing health care, education and wellbeing. Since this document was signed in the presence of 3,000 delegates from 134 countries and 67 nongovernmental organizations, the global rise of neoliberalism has resulted in the rapid marketization of health care, reductions in public expenditure, and the greater involvement of the private sector in public services ( Exworthy, 2008) and the WHO played an instrumental part in this transformation ( Navarro, 2008).

We view the Alma Ata Declaration of 1978 as perhaps the last effort, at the global stage, to call for and seek implementation of an expansive, community-organized, state-protected, rights-based approach to human health. Embroiled in peak Cold War ideological conflict (being held in Kazakhstan, then part of the USSR, was no accident), it was, no doubt, nearly immediately hemmed in by competing ideological policy frames advanced by the neoliberal Bamako Initiative, which forced individual clinics to purchase their own medicines and introduced fee for service models ( Hanson & Mcpake, 1993; Knippenberg *et al.*, 1997), and the rapid expansion of World Bank (WB) loans and International Monetary Fund (IMF)-driven austerity conditions ( Kentikelenis & Stubbs, 2023).

The pivot away from a human rights frame towards explicitly market allocated health services came with high costs. Despite significant economic growth, much of the Global South remained unable to invest the necessary resources to building effective, responsive and adequate health systems. The WB- and IMF-provided “development” loans and rapidly accruing debt left little “fiscal space” for national investments in basic services and provision of social and economic rights ( Siddiqui, 2012).

Under this regime, the Declaration’s ethos of ‘health for all’ has been replaced with ‘health insurance for all’ ( Pandey, 2018); health care for all who can pay. Fifty years later, principles of horizontality and social change outlined by the Alma Ata declaration are truly disturbing—some might even say radical—in the hegemonic global health prioritizing top-down, technologically driven health programs and market-based solutions to chronic health and social issues ( Holst, 2020). Numerous academics and practitioners have expressed the concern that contemporary global health is rapidly departing from the principles of the declaration, and that now, more than ever, we need ‘reinvigorated social justice–based on political and social movements—an uphill struggle, to be sure, but a healthy one indeed’ ( Birn, 2018). Reflecting on the concerns of invisibility in global health, the colonized state of both global health and bioethics, we suggest that the ethos of the Alma Ata Declaration could be reintroduced through the notion of disturbing bioethics and its central aim of challenging comforting yet heuristic solutions to chronic social and health care issues.

A range of important ‘disturbing’ bioethical contributions are needed to continue to make progress towards social justice in global health. The Alma Ata Declaration is by no means the end of this conversation. We believe that it is simply a potent example example and perhaps a beginning of a new conversation.

## Moving forward and looking back

Taken together, in pursuing the exercise in ‘comforting’ bioethics, we suggest that invisibility should be made central to normative action in accordance with the best practices in the field. More specifically, we suggest that funders and institutions could

increase the funding of studies of invisibility in various global health contexts to gain a better understanding of the problem;

establish local bottom-up collaborative partnerships to empower invisible communities and address invisible problems;

collect qualitative, quantitative or mixed-methods data and prepare it for evidence-based policymaking, with the goal of reaching national and international regulators; and

perform stakeholder analysis, review gaps in evidence concerning invisibility and identify topics for advocacy.

We believe that it is useful to understand global health invisibility as a product of global health political economy, or at least as a phenomenon unfolding in a political context. Accordingly, we would like to add three disturbing conceptual points to the list above, to make a grand total of seven:

- Politicize the emergence of global health invisibility and oppose the depoliticized operationalization of the term, further linking it with the notions of coloniality, precarity and neoliberalization
- Acknowledge that intersectional issues cannot be easily resolved by the vertical approaches that currently dominate the landscape of contemporary global health and applied global health bioethics
  Advocate for distributive justice and a more horizontal approach to the conduct of health interventions as initially proposed by the WHO Alma Ata declaration, prioritizing cooperation, resistance and solidarity beyond the market logic.

## Footnotes

^1^ That is, to say nothing of the patients or care providers embroiled in the workaday struggle to assuage suffering, prevent death, to heal.

^2^ Arguably, too slowly.
