# Supplementary material for: Invisibility in global health: A case for disturbing bioethical frameworks
Source: Wellcome Open Res. 2023 Dec 14;8:191. Originally published 2023 Apr 27. [Version 2] doi: 10.12688/wellcomeopenres.19346.2 (PMC10835101; doi:10.12688/wellcomeopenres.19346.2)
Supplement: Peer review history

# Reviewer report: Reviewer response for version 2

**DOI:** 10.21956/wellcomeopenres.22780.r71349

**Published:** 2024-02-01

**Authors:** Ahmad Ozair

**Affiliations:** 1 Bloomberg School of Public Health, Johns Hopkins University, Baltimore, Maryland, USA; 2 Faculty of Medicine, King George's Medical University, Lucknow, Uttar Pradesh, India

The authors have substantially improved upon their prior work and have constructively engaged with peer-review. This is now a more nuanced and careful argument for how we can think about the problems of invisibility in global health.

 The manuscript would potentially benefit from a summary figure (perhaps a causal diagram?) that synthesizes the arguments in the manuscript.

Does the article adequately reference differing views and opinions?

Partly

Are all factual statements correct, and are statements and arguments made adequately supported by citations?

Partly

Is the Open Letter written in accessible language?

Yes

Where applicable, are recommendations and next steps explained clearly for others to follow?

Yes

Is the rationale for the Open Letter provided in sufficient detail?

Yes

Reviewer Expertise:

Public Health, LMIC research, Global Health, Health Outcomes

I confirm that I have read this submission and believe that I have an appropriate level of expertise to confirm that it is of an acceptable scientific standard.

# Reviewer report: Reviewer response for version 2

**DOI:** 10.21956/wellcomeopenres.22780.r71350

**Published:** 2024-02-01

**Authors:** Adia Benton

**Affiliations:** 1 Department of Anthropology, Northwestern University, Evanston, Illinois, USA

My only comment for this much-improved essay addresses the link between the first sentence and the second. Is the reader to assume that 'overlooked, underappreciated, or ignored' reside under the rubric of 'invisibility'?

Does the article adequately reference differing views and opinions?

Yes

Are all factual statements correct, and are statements and arguments made adequately supported by citations?

Yes

Is the Open Letter written in accessible language?

Yes

Where applicable, are recommendations and next steps explained clearly for others to follow?

Not applicable

Is the rationale for the Open Letter provided in sufficient detail?

Partly

Reviewer Expertise:

Global health, infectious diseases, political economy, Black studies

I confirm that I have read this submission and believe that I have an appropriate level of expertise to confirm that it is of an acceptable scientific standard.

# Reviewer report: Reviewer response for version 1

**DOI:** 10.21956/wellcomeopenres.21431.r56647

**Published:** 2023-06-21

**Authors:** Adia Benton

**Affiliations:** 1 Department of Anthropology, Northwestern University, Evanston, Illinois, USA

This is a challenging essay; it appears that what’s at stake for the authors is how a range of inequalities and hierarchies are addressed in the fields and practices of public health and bioethics. Attempts to redress problems that are located within marginalized or precarious groups is to characterize them as invisible -- thus the intervention requires visibilizing for the sake of intervention. But the authors suggest that even as such inequalities may serve as the engine and fuel for a range of political, social and economic interventions for health, these same interventions might also produce and conceal those (and emergent) inequalities, sheltering them from view.

 More importantly, they do not address what lies at the root of invisibility. Throughout, I found myself wondering: What analytical and empirical weight should be afforded to ‘invisibility’ as an organizing principle, when ignorance, concealment, etc have all been theorized in relation to similar power dynamics? Is it worth taking on this 'native' concept within global health? While the heuristic may help these analysts move beyond the descriptive (what is invisibility–though maybe the question is really ‘what is invisible’?), toward practice and process (‘how is invisibility made and unmade in practice’), I thought the authors called for something a bit more elemental: attention to the subjects and objects of in/visibility. To suggest that something is invisible is to also suggest an object and a subject of ocular perception, a visual aesthetics of power, a regime of visibility are all at play. Thus, attention to invisibility is a methodological and analytical move, guiding the transdisciplinary examinations alluded to in the letter’s introduction: who and what is doing the perceiving, what is being perceived and ‘apprehended’ (in many senses of the word)? What is surfaced, concealed, and sublimated, through which means and to what end? What is at stake – and for whom –in those concealments, sublimations and surfaces? And are these signs and secrets of the global health landscape only to be recognized and decoded by a certain set of social and cultural analysts via a specific set of conceptual frames? (This is a Simmelian argument, I think). I know that the tendency within global health critique is to offer solutions, answers, alternatives (and even some concessions) – and Alma Ata, as well as the disturbing/comforting framework, which reflects a general discomfort with liberal reform, have been offered in this critical position. But I wondered what would happen if the authors instead posed, irrespective of the disturbing and comforting effects of specific interventions, the elemental questions raised above: what has been settled as seen, what is to be unsettled and unseated, such that crucial underlying issues, hitherto ignored or concealed social and political problems, come to the surface?

 I think that answering these question might have an impact on the first few recommendations offered. I also think that the 'disturbing' recommendations could be better integrated in the analysis in the earlier segments of the letter. Why not shake things up from the beginning?

Does the article adequately reference differing views and opinions?

Yes

Are all factual statements correct, and are statements and arguments made adequately supported by citations?

Yes

Is the Open Letter written in accessible language?

Yes

Where applicable, are recommendations and next steps explained clearly for others to follow?

Not applicable

Is the rationale for the Open Letter provided in sufficient detail?

Partly

Reviewer Expertise:

Global health, infectious diseases, political economy, Black studies

I confirm that I have read this submission and believe that I have an appropriate level of expertise to confirm that it is of an acceptable scientific standard, however I have significant reservations, as outlined above.

# Response

**Published:** 2023-11-22

**Authors:** Arsenii Alenichev

**Affiliations:** University of Oxford, Oxford, England, UK

**Dear Adia Benton,**

**Thank you very much for an opportunity to make the article stronger. We would like to sincerely apologize for the substantial delay: many of us got caught in the cycle of deadlines, much-needed holidays and other work commitments. It took us a really long time to reconnect about this conceptual article, and think through your insightful comments. We did our best to address your comments. Please let us know if there is anything else we can do to make it better, and again, sorry for such a delay. Here goes the summary of changes:**

 This is a challenging essay; it appears that what’s at stake for the authors is how a range of inequalities and hierarchies are addressed in the fields and practices of public health and bioethics. Attempts to redress problems that are located within marginalized or precarious groups is to characterize them as invisible -- thus the intervention requires visibilizing for the sake of intervention. But the authors suggest that even as such inequalities may serve as the engine and fuel for a range of political, social and economic interventions for health, these same interventions might also produce and conceal those (and emergent) inequalities, sheltering them from view.  

**Thank you for this generous summary.**

 More importantly, they do not address what lies at the root of invisibility. Throughout, I found myself wondering: What analytical and empirical weight should be afforded to ‘invisibility’ as an organizing principle, when ignorance, concealment, etc have all been theorized in relation to similar power dynamics?  

**This is a fantastic point, and something that we have tried to expand our analysis of visibility to include. We have now included the following paragraph:**

**The notion of visibility’ or ‘invisibility’ as a category of analysis within the social sciences (Brighenti 2007) and studies of global health is not new (Brown et al. 2012; Brandt 2013). Visibility has been studied as a field of power, condensed and deployed by states and academic disciplines to manage and control populations, problems, and knowledge respectively (Foucault 1972; 1973; Scott 1998). Within global health, the notion of visibility has been theorized as the political construction and mobilization of justificatory and explanatory metrics, quantitative and qualitative facts in global health governance (Adams et al 2016; Benton 2012). Subaltern and postcolonial studies have long emphasized the partial, positioned, and power-laden relationships that enable certain views, conceptions, and thus unequal knowledge and often reproduction of dominating relationships and inequality (Grosfoguel 2002; Grosfoguel 2007). Scholarly claims of “invisibility” in global health may also be related to the linked political and moral economies of ignorance (Kourany and Carrier 2020), secrecy, concealment, and hiding (Jones 2014; Scott 2009: 179-219), as well as social hierarchies of recognition and their relationship to legibility and care (Benton 2015: 97-113). As this diverse social scientific literature demonstrates, the concepts of visibility and invisibility are inseparable from a normatively contested and highly unequal political economy that shapes the terrain of global health practice.**

 Is it worth taking on this 'native' concept within global health?  

**Yes, we think it is – but perhaps in another paper. We have gestured to its need to be denaturalized in the paragraph above.**  

 While the heuristic may help these analysts move beyond the descriptive (what is invisibility–though maybe the question is really ‘what is invisible’?), toward practice and process (‘how is invisibility made and unmade in practice’), I thought the authors called for something a bit more elemental: attention to the subjects and objects of in/visibility. To suggest that something is invisible is to also suggest an object and a subject of ocular perception, a visual aesthetics of power, a regime of visibility are all at play. Thus, attention to invisibility is a methodological and analytical move, guiding the transdisciplinary examinations alluded to in the letter’s introduction: who and what is doing the perceiving, what is being perceived and ‘apprehended’ (in many senses of the word)? What is surfaced, concealed, and sublimated, through which means and to what end? What is at stake – and for whom –in those concealments, sublimations and surfaces?  

**Thank you for this incredibly thoughtful interpretation of the essay and its intervention. We agree with you, though you’ve captured it here in a much clearer and more potent way.**

 And are these signs and secrets of the global health landscape only to be recognized and decoded by a certain set of social and cultural analysts via a specific set of conceptual frames? (This is a Simmelian argument, I think).  

**This a very good point – our intention was not to imagine some delimited set critical theory-competent high priests capable of decoding the rightness or wrongs of various forms of occlusions and visions. Our intervention, particularly with the ‘comforting/disturbing’ frames is to actually try to disrupt the existing boundary work done within contemporary ‘global’ bioethics, and in fact expand the range of considerable and considered forms of method, analysis, critique, political action, practice.**

 I know that the tendency within global health critique is to offer solutions, answers, alternatives (and even some concessions) – and Alma Ata, as well as the disturbing/comforting framework, which reflects a general discomfort with liberal reform, have been offered in this critical position. But I wondered what would happen if the authors instead posed, irrespective of the disturbing and comforting effects of specific interventions, the elemental questions raised above: what has been settled as seen, what is to be unsettled and unseated, such that crucial underlying issues, hitherto ignored or concealed social and political problems, come to the surface?  

**We think that this would be a fantastic follow on essay building from the initial intuitions presented here.**

 I think that answering these question might have an impact on the first few recommendations offered. I also think that the 'disturbing' recommendations could be better integrated in the analysis in the earlier segments of the letter. Why not shake things up from the beginning?

# Reviewer report: Reviewer response for version 1

**DOI:** 10.21956/wellcomeopenres.21431.r59328

**Published:** 2023-06-21

**Authors:** Ahmad Ozair

**Affiliations:** 1 Bloomberg School of Public Health, Johns Hopkins University, Baltimore, Maryland, USA; 2 Faculty of Medicine, King George's Medical University, Lucknow, Uttar Pradesh, India

The authors have submitted an insightful and thought-provoking open letter on invisibility in global health, wherein they highlight the 'comforting' and 'disturbing' frameworks needed by global health bioethics. 

 While the work is interesting, it should be revised further. Given below are my comments in no particular order:

1. Several of the statements in the manuscript need citations but are not supported by any. This issue exists throughout the manuscript but is especially problematic with regard to the "Encountering global health invisibility: a few empirical snapshots" section where all the examples provided need references. These references may be non-academic in nature as well.
2. The manuscript's statement of "we call for taking seriously" implies the rest of the academic world has not taken this seriously at all in the face of a substantial global reflection on these considerations.
  I would recommend authors make the language less boisterous and more academic so that readers can focus on the argument itself. 
3. I do not fully agree with several of the recommendations that the manuscript makes, given that little quantitative data has been provided to demonstrate that the recommendations provide substantial global health returns on the time, energy, and funds invested.
  I am particularly concerned by the strong recommendation made for 'politicizing global health' efforts (invisibility alleviation), particularly since the current version of the manuscript does not provide evidence to support this recommendation. 
  While in evidence-based medicine, we are utilizing the GRADE approach to go from evidence to decision (EtD) (i.e., recommendations), here, the manuscript states several recommendations without providing evidence, let alone critically appraising it or performing quantitative synthesis. 

Does the article adequately reference differing views and opinions?

Partly

Are all factual statements correct, and are statements and arguments made adequately supported by citations?

Partly

Is the Open Letter written in accessible language?

Yes

Where applicable, are recommendations and next steps explained clearly for others to follow?

Yes

Is the rationale for the Open Letter provided in sufficient detail?

Yes

Reviewer Expertise:

Public Health, LMIC research

I confirm that I have read this submission and believe that I have an appropriate level of expertise to confirm that it is of an acceptable scientific standard, however I have significant reservations, as outlined above.

# Response

**Published:** 2023-11-22

**Authors:** Arsenii Alenichev

**Affiliations:** University of Oxford, Oxford, England, UK

**Dear Ahmad Ozair,**

**Thank you very much for an opportunity to make the article stronger. We would like to sincerely apologize for the substantial delay: many of us got caught in the cycle of deadlines, much-needed holidays and other work commitments. It took us a really long time to reconnect about this conceptual article, and think through your insightful comments. We did our best to address your comments. Please let us know if there is anything else we can do to make it better, and again, sorry for such a delay. Here goes the summary of changes:**

 The authors have submitted an insightful and thought-provoking open letter on invisibility in global health, wherein they highlight the 'comforting' and 'disturbing' frameworks needed by global health bioethics.   

**Thank you for this supportive comment!**

 While the work is interesting, it should be revised further. Given below are my comments in no particular order:

1. Several of the statements in the manuscript need citations but are not supported by any. This issue exists throughout the manuscript but is especially problematic with regard to the "Encountering global health invisibility: a few empirical snapshots" section where all the examples provided need references. These references may be non-academic in nature as well.
**Thank you for this important feedback. We have added a new paragraph and significantly expanded our theoretical survey of ‘visibility’ as an analytic category within socials sciences and global health. For instance, we have added this:  **
** The notion of visibility’ or ‘invisibility’ as a category of analysis within the social sciences (Brighenti 2007) and studies of global health is not new (Brown et al. 2012; Brandt 2013). Visibility has been studied as a field of power, condensed and deployed by states and academic disciplines to manage and control populations, problems, and knowledge respectively (Foucault 1972; 1973; Scott 1998). Within global health, the notion of visibility has been theorized as the political construction and mobilization of justificatory and explanatory metrics in global health governance (Adams et al 2016). Subaltern and postcolonial studies have long emphasized the partial, positioned, and power-laden relationships that enable certain views, conceptions, and thus unequal knowledge and often reproduction of dominating relationships and inequality (Grosfoguel 2002; Grosfoguel 2007).**
2. The manuscript's statement of "we call for taking seriously" implies the rest of the academic world has not taken this seriously at all in the face of a substantial global reflection on these considerations.
  I would recommend authors make the language less boisterous and more academic so that readers can focus on the argument itself.
**Thank you for this suggestion, we agree with you. We have scratched the “we call for” language.  **
3. I do not fully agree with several of the recommendations that the manuscript makes, given that little quantitative data has been provided to demonstrate that the recommendations provide substantial global health returns on the time, energy, and funds invested.
**Thank you for this perspective. We believe that it is ok to disagree on these points (partly, to our understanding, this is what the purpose of these Wellcome letters are for!). But, providing an econometric analysis of the outcomes of our proposed recommendations is not within scope of this particular piece. We will consider doing this in a potential follow up article.  **
4. I am particularly concerned by the strong recommendation made for 'politicizing global health' efforts (invisibility alleviation), particularly since the current version of the manuscript does not provide evidence to support this recommendation. 
**Reflecting on numerous social studies of Global health, we would like to highlight the fact that it has already been politicized (how could it not be?). From its global institutional forms, policies advanced or declined, modes of financing pursued, ideas and enforcement of human rights or market allocation of crucial health goods and services, structures of global debt and state-level fiscal allocation to basic service provision and care, to forms of scholarship and the views and perspectives privileged to be counted as evidence-based or scientific. This is all political. We are calling for a recognition that this is the case and perhaps this is disturbing.   While in evidence-based medicine, we are utilizing the GRADE approach to go from evidence to decision (EtD) (i.e., recommendations), here, the manuscript states several recommendations without providing evidence, let alone critically appraising it or performing quantitative synthesis.  Thank you, but we will not be able to incorporate that point into this brief letter piece. We might explore it in a follow up article.**

# Reviewer report: Reviewer response for version 1

**DOI:** 10.21956/wellcomeopenres.21431.r59329

**Published:** 2023-06-19

**Authors:** Sridhar Venkatapuram

**Affiliations:** 1 Philosophy, University of Johannesburg, Auckland Park, Gauteng, South Africa; 2 Global Health Institute, Population Health Sciences, King's College London, London, England, UK

This open letter seeks to report on a 2022 meeting on the topic of invisibility in global health, and then present a manifesto of sorts on how funders and institutions should act in light of the identification of the 'invisibility' phenomenon in global health. Before the 7 point call to action, the authors first link three different ideas. First, they seek to establish through 5 case studies (empirical data) that there are diverse kinds of invisibility in global health. Second, they posit that addressing the complex and deep rooted sources of global health invisibility requires an analytical distinction and two pronged approach entailing "comforting" and "disturbing" bioethics. Third, the authors then assert that global health goals of the 21st century would be more likely to be achieved through a disturbing bioethics, and such an effort should be motivated by the ethos of the WHO Alma Ata declaration of 1978.

 I will first address some minor points and then offer some broader thoughts.

1. A direct ref to the Alma Ata declaration is needed so that readers can see what the document actually said. Other writers have commented on what they think the declaration said, what has gone wrong since, etc. 
2. It would be good to see explicitly what conception of global health is being used in this letter. The refs in the first para are all about health concerns in LMICs.
3. It is not clear where in the letter discussion moves from reporting on a meeting to making an argument based on the discussion at the meeting. It's a bit awkward trying to see if the reasoning being presented is record of the meeting, or an argument being presented on the basis of the meeting. Reviewing a meeting record is different that reviewing analyses or argument.

On a more general level, I am clear about the first of the 3 points, but less convinced about the second parts. 

1. "Invisibility" is not new to global health. If we are to go along with the idea that HIV/AIDS invented global health (Brandt 2013) then we have to recall that silence=death was a call to arms to fight against invisibility, shame, social marginalization, willful blindness, purposeful erasure, minimization, neglect, etc. Even before HIV/AIDS activism, historians such as those building " subaltern studies" have argued for dismantling grand narratives and big man histories to tell the stories of the subaltern. Given the authors argue for trans-disciplinary examination of invisibility in global health it is slightly odd to not have some acknowledgement of important efforts in global health as well as other fields.
2. It is unclear why or how invisibility in global health is to be addressed through "global health bioethics"? What is this global health bioethics and what is its significance in global health? As the authors are recommending actions for funders and institutions, is global health bioethics the tool or language these actors most often use, or something else?
3. There is a tension in the letter which asserts that "ethicality can be achieved when power structures are challenged and reassembled in a structurally competent way." And then the authors make recommendations to just such funders and institutions to challenge themselves and reassemble them selves. 
4. There is an over idealization of the Alma Ata declaration of 1978. One could argue that the AA declaration sought to achieve sufficiency/ or apply a basic needs approach to health. Every citizen would receive a basic package of healthcare accessed through local health centres. There is much to be valued in a such an approach and also much to be skeptical about. How did the countries that applied the AA declaration address HIV/AIDS, disability, women's health, and other health issues that were not linked to a profile of an "average citizen". 
5. The letter does not make any reference to the Astana Declaration of 2018 which was aimed to be a 40 year follow up to the AA Declaration. It will be clear that the Astana Declaration focuses on Primary Health Care, and that is because we now recognize that the AA declaration was really focused on one level of health care provision. What this means is that all the spaces of invisibility in contemporary global health, that the authors are concerned about, will not be sufficiently addressed by the ethos of the AA declaration which is really focused at the local service delivery level. 
6. Rather than just a primary health care framework and ethics of the local, what would better serve making all forms of health injustice visible is a theory that gives moral significance to every individual's health and wellbeing, and which has a scope from the local to the global. The ethics of AA declaration had limited scope and may be silent on various forms of injustice we now recognize.

Does the article adequately reference differing views and opinions?

No

Are all factual statements correct, and are statements and arguments made adequately supported by citations?

Partly

Is the Open Letter written in accessible language?

Yes

Where applicable, are recommendations and next steps explained clearly for others to follow?

Partly

Is the rationale for the Open Letter provided in sufficient detail?

Partly

Reviewer Expertise:

Global health, public health, health inequalities, applied ethics, political philosophy, social justice theories, health ethics, public health ethics, development theory.

I confirm that I have read this submission and believe that I have an appropriate level of expertise to confirm that it is of an acceptable scientific standard, however I have significant reservations, as outlined above.

# Response

**Published:** 2023-11-22

**Authors:** Arsenii Alenichev

**Affiliations:** University of Oxford, Oxford, England, UK

**Dear Sridhar Venkatapuram,**

**Thank you very much for an opportunity to make the article stronger. We would like to sincerely apologize for the substantial delay: many of us got caught in the cycle of deadlines, much-needed holidays and other work commitments. It took us a really long time to reconnect about this conceptual article, and think through your insightful comments. We did our best to address your comments. Please let us know if there is anything else we can do to make it better, and again, sorry for such a delay. Here goes the summary of changes:**

 This open letter seeks to report on a 2022 meeting on the topic of invisibility in global health, and then present a manifesto of sorts on how funders and institutions should act in light of the identification of the 'invisibility' phenomenon in global health. Before the 7 point call to action, the authors first link three different ideas. First, they seek to establish through 5 case studies (empirical data) that there are diverse kinds of invisibility in global health. Second, they posit that addressing the complex and deep rooted sources of global health invisibility requires an analytical distinction and two pronged approach entailing "comforting" and "disturbing" bioethics. Third, the authors then assert that global health goals of the 21st century would be more likely to be achieved through a disturbing bioethics, and such an effort should be motivated by the ethos of the WHO Alma Ata declaration of 1978.

 I will first address some minor points and then offer some broader thoughts.

1. A direct ref to the Alma Ata declaration is needed so that readers can see what the document actually said. Other writers have commented on what they think the declaration said, what has gone wrong since, etc. 
**Thank you for addressing this omission. We have now included a citation to the Alma Ata declaration.  **
2. It would be good to see explicitly what conception of global health is being used in this letter. The refs in the first para are all about health concerns in LMICs.
**Thanks for this helpful feedback. We’ve added a citation to Farmer et al’s *Reimagining Global Health* and clarified that we mean that the ‘academic discipline’ of global health is what we are referring to when we refer to ‘invisibility in global health.’**
3. It is not clear where in the letter discussion moves from reporting on a meeting to making an argument based on the discussion at the meeting. It's a bit awkward trying to see if the reasoning being presented is record of the meeting, or an argument being presented on the basis of the meeting. Reviewing a meeting record is different that reviewing analyses or argument.
**Thanks for this great point- we understand this confusion, or lack of clarity. We have tried to improve the language throughout to make clear that this is not a record of the meeting, but rather an analysis of shared empirics and an argument presented based on those presentations. ** 

On a more general level, I am clear about the first of the 3 points, but less convinced about the second parts. 

1. "Invisibility" is not new to global health. If we are to go along with the idea that HIV/AIDS invented global health (Brandt 2013) then we have to recall that silence=death was a call to arms to fight against invisibility, shame, social marginalization, willful blindness, purposeful erasure, minimization, neglect, etc. Even before HIV/AIDS activism, historians such as those building " subaltern studies" have argued for dismantling grand narratives and big man histories to tell the stories of the subaltern. Given the authors argue for trans-disciplinary examination of invisibility in global health it is slightly odd to not have some acknowledgement of important efforts in global health as well as other fields.
**These are fantastic points, and we have tried to expand our discussion of past scholarship and theorization of visibility in global health by adding a new paragraph that offers at least a gloss of these important contributions. Currently, the new paragraph reads:   The notion of visibility’ or ‘invisibility’ as a category of analysis within the social sciences (Brighenti 2007) and studies of global health is not new (Brown et al. 2012; Brandt 2013). Visibility has been studied as a field of power, condensed and deployed by states and academic disciplines to manage and control populations, problems, and knowledge respectively (Foucault 1972; 1973; Scott 1998). Within global health, the notion of visibility has been theorized as the political construction and mobilization of justificatory and explanatory metrics in global health governance (Adams et al 2016). Subaltern and postcolonial studies have long emphasized the partial, positioned, and power-laden relationships that enable certain views, conceptions, and thus unequal knowledge and often reproduction of dominating relationships and inequality (Grosfoguel 2002; Grosfoguel 2007).**
2. It is unclear why or how invisibility in global health is to be addressed through "global health bioethics"? What is this global health bioethics and what is its significance in global health? As the authors are recommending actions for funders and institutions, is global health bioethics the tool or language these actors most often use, or something else?
**We would like to highlight that we think that global health bioethics, as an explicitly normative discipline, has an even stronger opportunity than basic empirical social science (and perhaps even an obligation) to engage not just with the moral possibilities and consequences of global health programs, policies, actions, and plans, but to also consider, comment on, and seek to shape the normative content, structure, and methods of production of knowledge within the discipline itself.   For this reason, this intervention is a plea for the discipline to not simply stay blinkered by what is institutionally rewarded and may feel “comfortable” for scholars, particularly in the Global North, but to also incorporate and explore ways of knowing, forms of knowledge, and political engagements that may challenge those ways of knowing and doing business. This goes beyond normalized institutionalized efforts and, in our opinion, it is about seeking to attend to and in fact value modes of bioethical research and practice that may be institutionally disturbing.  **
3. There is a tension in the letter which asserts that "ethicality can be achieved when power structures are challenged and reassembled in a structurally competent way." And then the authors make recommendations to just such funders and institutions to challenge themselves and reassemble them selves. 
**We would like to highlight the broader scope: we live in a moment of upheaval, contentious politics, social movements, and organized action. Perhaps this is intended as a warning shot across the bow.  **
4. There is an over idealization of the Alma Ata declaration of 1978. One could argue that the AA declaration sought to achieve sufficiency/ or apply a basic needs approach to health. Every citizen would receive a basic package of healthcare accessed through local health centres. There is much to be valued in a such an approach and also much to be skeptical about. How did the countries that applied the AA declaration address HIV/AIDS, disability, women's health, and other health issues that were not linked to a profile of an "average citizen". 
**We agree that we need to make the motivation for our focus and attention on the Alma Ata declaration clearer. We have included the following paragraph:   We view the Alma Ata declaration as the perhaps last effort at the global stage to call for and seek implementation of an expansive, community-organized, state-protected, rights-based approach to human health. Embroiled in peak Cold War ideological conflict (being held in Soviet Kazakhstan was no accident), it was, no doubt, nearly immediately hemmed in by competing ideological frames such as the neoliberal Bamako Initiative (which forced individual clinics to purchase their own medicines and introduced fee for service models) (Knippenberg 1997; ) and the rapid expansion of World Bank loans and IMF-driven austerity conditions (Kentikelenis and Stubbs 2023).  **
5. The letter does not make any reference to the Astana Declaration of 2018 which was aimed to be a 40 year follow up to the AA Declaration. It will be clear that the Astana Declaration focuses on Primary Health Care, and that is because we now recognize that the AA declaration was really focused on one level of health care provision. What this means is that all the spaces of invisibility in contemporary global health, that the authors are concerned about, will not be sufficiently addressed by the ethos of the AA declaration which is really focused at the local service delivery level. 
**We understand that the spirit of the Alma Ata declaration continues to be invoked in drips and drabs, but we believe that declarations in this spirit, such as the Astana Declaration, continue to leave absent the ‘disturbing’ fact of the necessity of redistributive financing mechanisms to achieve any of their noble goals. In our view, this is part of the need for a disturbing bioethics, to perhaps name the normative and moral content of what is too often assumed a neutral, level political economic playing field.  **
6. Rather than just a primary health care framework and ethics of the local, what would better serve making all forms of health injustice visible is a theory that gives moral significance to every individual's health and wellbeing, and which has a scope from the local to the global. The ethics of AA declaration had limited scope and may be silent on various forms of injustice we now recognize.
**We agree with you – a range of important ‘disturbing’ bioethical contribution are needed to continue to make progress towards social justice in global health. The Alma Ata Declaration is no means the end of this conversation, simply an example and perhaps a beginning. We have added a couple lines to this effect.**
